# Modeling Limited-Stop Bus Corridor Services with Fare Payment Mode Choice and Trip Purpose Consideration

**DOI:** 10.1155/2022/4329943

**Published:** 2022-11-02

**Authors:** Chunyan Tang, Ying-En Ge, Jiyu Zhang, Qi Xu

**Affiliations:** ^1^College of Transportation Engineering, Dalian Maritime University, Dalian 116026, Liaoning, China; ^2^College of Transportation Engineering, Chang'an University, Xi'an, Shaanxi, China; ^3^Key Laboratory of Intelligent Transportation in Guangxi Zhuang Autonomous Region, Guilin 541004, China

## Abstract

This paper proposes a novel model for optimizing limited-stop bus corridor services with consideration of varied payment modes and different trip purposes. In the proposed model, the bus dwell time at a stop is dependent on the fare payment modes and the number of passengers getting off and waiting at the stop while those with the similar trip purpose are grouped into one user class. Given an origin–destination (OD) passenger trip matrix and a set of candidate bus lines serving a corridor, the proposed model is to minimize the total social cost that consists of the cost to the bus operator and the cost to the passengers. In the formulation of the optimization problem, a weighting parameter is adopted to balance the operator cost and the passenger cost. Numerical examples are presented to illustrate the importance of considering passenger flow impacts on bus (and passenger) travel times in the proposed model. We also investigate effects on the optimal limited-stop services (e.g., short-turn, skip-stop, and express) taking into account the choice of fare payment modes (e.g., on-board fare collection including payment by cash, magnetic strip or smart card, off-board fare collection) and different values of travel time due to passenger trip purposes. It is shown that the off-board payment mode would be more efficient in a high-demand corridor, that more passengers prefer to express and skip-stop services rather than normal regular services in the four collection systems, and that different limited-stop service plans should be used for different periods of the day in response to temporal variation in OD passenger travel patterns. The intellectual merit of this paper is not the seemingly obvious conclusions but that the proposed model can handle the problem of limited-stop bus corridor service design with the consideration of fare payment mode choice and trip purposes.

## 1. Introduction

The transit network design problem (NDP) is of great importance to transit planning, either strategic or tactical or operational [1, 2]. In fact, it is a basic and critical determinant that can significantly influence the other components of transit planning. The transit network design problem relies heavily on methods for capturing passengers' behavior of route choice of all available alternatives serving their origins and destinations. The output of the transit NDP includes an optimal set of bus routes and their frequencies [3, 4]. Since the passenger demand distribution along a bidirectional bus corridor is usually neither uniform nor symmetric, it has been proposed [5] that the bus corridor operational plan may not have to offer the same services at every stop but can tailor the services to the market in a more efficient way, and the proposed schemes include zonal express service, short turning service, restricted or semirestricted zonal service, deadheading service, and limited-stop zonal service. Intuitively, a stop with high demand may well deserve a higher service frequency than those stops with lower demand. Therefore, bus line stop service schemes may vary, e.g., visiting all or a subset of stops along a public transit corridor with different frequencies, which provides a significant way to reduce the operational cost to the service provider and the passengers' travel times (or equivalently time cost). The savings of the cost to the operator can be attributed to the less number of required buses while the service users are benefited due to less waiting or dwell times at stops and shortened journey times. It is these benefits that attract more and more attention to investigate these bus service schemes [6–8].

In this paper, the design of limited-stop bus corridor services is considered. The strategy of limited-stop services is suitable for longer corridors and not like the other aforementioned schemes due to the fact that it has no requirements on the level of the ratio of the corridor's peak volume (PV) to its uptown boardings (UB) or uptown alightings for the outbound direction in the p.m. peak, which is termed PV/UB ratio. According to Furth and Day [5], “a limited-stop pattern has a service zone in which passengers may freely board and alight at any stop” whereas “outside the service zone, buses stop only at designated stops.” “At these designated stops, passengers may both board and alight.” Different from the descriptive summary or discussion in Furth and Day [5], Leiva et al. [9] offer a series of normative models for the design of limited-stop services for an urban bus corridor with capacity constraints. Another difference between these two previous related papers lies in their definitions of limited-stop services, which may be due to their different scenarios of interest, which respectively arise from the practice in the United States of America and in Chile. Specifically, different from the definition above in Furth and Day [5], Leiva et al. [9] simply define “limited-stop services” as “services that visit only a subset of the stops on a route.” In fact, the definition of limited-stop service by Furth and Day is a special case of Leiva et al's definition. This paper follows the definition of the concept in Leiva et al. [9] and carries on to investigate the effects of fare payment modes and trip purposes on the resulting design of the limited-stop bus services.

## 2. Literature Review

### 2.1. Overview of Limited-Stop Services

Previous transit studies on the design of bus service schemes can be classified into three groups, i.e., a combination of short-turn service and full-stop (FS) service, a combination of skip-stop service and FS service, and a combination of multiple service patterns such as FS, short-turn, skip-stop, and express [10]. Short-turn, skip-stop, and express services can be collectively referred to as limited-stop services that visit only a subset of the stops on a route [9].

The first group of studies aims to determine a combinational service scheme of short-turn service and FS service. It is particularly useful for a bus route with a high passenger demand concentration on some segments because of increasing service frequencies on these high load route segments for accommodating the imbalanced demand pattern. Delle Site and Filippi [11] proposed an optimization model to determine frequencies of FS and short-turn for a given corridor operation. This model produces trade-offs between users' and operator's costs. Ceder and Israeli [6] described a procedure, based on a deficit function theory, to design short-turn trips by deleting unnecessary departure times in a given timetable. Ulusoy and Chien [12] introduced express service into the FS and short-turn services, using a logit-based model to estimate ridership with these services. Moon et al. [13] investigated the implementation of short-turn services on multiple crowding routes in a transit network and decided the routes, turn-back points, and fleet sizes of short-turn services by minimizing the total system cost.

The second group of studies aims to determine a combinational service scheme of skip-stop service and FS service. Its services stop with high demand more frequently than those stops with lower demand, and thus, it is useful for transit routes with a high concentration of trips in few origin–destination pairs. Liu et al. [14] investigated the design of robust skip-stop service based on travel time variations. Considering both vehicle overtaking and time-dependent stochastic travel time, Wu et al. [15] developed a simulation-based robust optimization methodology for determining the skip-stop scheme. Soto et al. [16] explored the possibility of modeling passenger assignment as a stochastic process and then designed robust skip-stop services. Without predetermined a set of stops served a skip-stop service, Wang et al. [17] developed a mathematical model to determine the set of bus stops served by the skip-stop service, operation frequencies, and fleet size assignment while considering passengers' service choices. From the perspective of user's comfort, García-Albarracín and Jaramillo-Ramírez [18] proposed a methodology to minimize the peak load profile, finding the set of stops for the skip-stop service and the fleet split. Liang et al. [19] considered the trade-off between the users and operator to formulate a multiobjective optimization problem. Suman et al. [20] investigated the risks involved in oversimplifying a skip-stop service design problem by implicitly assuming that passengers behave altruistically and found that it often underestimates the frequencies required for the system to satisfy its demand.

The first two groups of studies above show that an additional service pattern (e.g., short-turn or skip-stop) can further improve the transit service results given a particular demand pattern. However, currently, most of the transit routes are characterized by mixed and complex demand patterns. Thus, there is a need to better accommodate this fluctuating demand by using a combination of multiple service patterns [7]. Leiva et al. [9] proposed an optimization approach to examine which of given service patterns consisting of skip-stop, full-stop, short-turn, and express, should be provided at what frequencies with a given origin–destination demand matrix. Based on this model proposed by Leiva et al. [9], Larrain et al. [21] considered different characteristics of demand on a bus corridor, including the base load profile shape, the scale of demand, and the demand imbalance between outbound and inbound directions. Later, considering transfer demand elasticity, Ulusoy and Chien [12] investigated the determination of service patterns by minimizing the user cost and the operator cost. In the recent study, Tang et al. [22] proposed a new network-based methodology for optimizing multiple service patterns, based on a complete timetable using the full-stop pattern.

Extensive research has been conducted to investigate the use of multiple service patterns in improving bus operating efficiency and service levels, but with little attention so far to the effects of fare payment modes and trip purposes on the design of multiple service patterns.

Fare payment modes would significantly affect bus dwell times at stops and hence the costs to the operator and to the passengers [23, 24]. Fare payment modes in the literature include payment outside buses, a prepaid card validated inside buses, and cash transactions. Tirachini [24] showed that “substantial time savings are accruable if payment methods are upgraded from slow techniques, such as cash transactions to the fastest one (fare paid outside buses) while intermediate technologies such as prepaid cards validated inside buses (with or without contact) fall in between.” This investigation supports metro/underground systems and BRT systems to adopt the method of fare collection outside buses or carriages. The fastest fare payment mode may incur a considerable cost or require a significant amount of investment on facility improvement. However, as the new technologies are more applicable for payment outside buses, such as smartphone apps, or Internet, the off-payment mode has become less costly for use in collecting bus fare. Tirachini and Hensher [23] took into account the fare payment system as a policy variable that has a great influence over passenger travel times and operator cost and compare the performance of four alternative payment modes (cash, magnetic strip, contactless card, and off-board). We consider the fare payment system as a policy factor within the limited-stop bus corridor service framework to investigate its impacts on bus dwell times at stops and hence on the resulting service plan, which is an intellectual merit of this paper.

In practice, values of travel times usually vary with user trip purposes. For example, compared with those trips for work, passengers for shopping or leisure generally have lower values of travel times. It is reasonable to argue that it may not be efficient to apply the same bus service plan for the whole day, the reason being that the constituents of passenger flows generally vary in different periods of a day in terms of trip purposes. Most of passengers travel for commuting in the morning or evening peak, and most of the passengers in off-peak periods travel for such purposes as shopping, leisure, etc. Hence, it is of great importance for us to consider passenger trip purposes while optimizing bus corridor services. In this paper, we aim to extend the modeling framework of Leiva et al. [9] and investigate the design of limited-stop services for passengers with multiple trip purposes, which results in multiclass limited-stop bus corridor service models. Moreover, values of travel times may vary due to many other factors, such as passenger income level, access distance, and departure time (e.g., morning peak time, lunch time, or evening peak time). But, in this paper, we focus on the variation in the value of time due to different passenger trip purposes.

### 2.2. Contributions and Organization

The contributions of this study are threefold. First, we consider the fare payment system as a policy factor within the limited-stop bus corridor service framework to investigate its impacts on bus dwell times at stops and hence on the resulting service scheme. Second, previous literature on transit services design problems does not consider the effects of passenger trip purposes, and values of travel times are given as constant without distinguishing passengers' trip purposes (e.g., [9]). To the best of our knowledge, this study appears to be the first study that addresses the design of limited-stop services by trip purposes, which yields a multiclass passenger flow limited-stop bus corridor service model. Third, the model proposed in this paper is to minimize a weighted sum of the bus operator cost plus passenger (or user) cost, in which a weighting parameter is introduced for the planner to balance the interests of the bus operator and users.

The remainder of this paper is organized as follows.  Section 3 systematically describes the representation of bus corridor stop services and presents a limited-stop services design model with dwelling time at stops, in which fare payment modes and trip purposes are captured. We present in Section 4 an in-depth analysis of the numerical results generated by applying the proposed model to an example bus corridor.  Section 5 closes the paper with some concluding remarks.

## 3. Model Formulation

### 3.1. Assumptions

Given a fixed origin–destination (OD) matrix of passenger trips, this paper considers various bus lines and their frequencies, each of which may serve only a subset of the bus stops along a bidirectional public transit corridor. It is assumed that the bus dwell time at a stop depends on passenger boarding and alighting flows plus fare payment modes used. In addition, decelerating and accelerating times before or after a stop are treated as part of the bus travel time between two successive stops, which is assumed to be fixed and given in this paper. Although passengers randomly arrive at their respective origin stops, they are assumed to reach a constant average rate given by the passenger OD matrix. Under the assumption that a passenger always chooses the best stop to transfer, passenger assignment reflects the existence of a set of attractive itinerary segments for each OD pair that minimizes their expected travel time. This paper does not take into account budget constraints and/or vehicular traffic flow-dependent congestion but bus capacity constraints and/or in-vehicle crowding, and corridor constraints are considered. It is also assumed that all passengers will pay the same fare on each line, so the total user fare is constant and hence not included in the objective function of the proposed model.

In order to formulate the methodology as a programming model, the parameters and variables are defined in the Notation of Appendix.

### 3.2. Operator Cost

The operator's total cost of a bus corridor can be divided into two components: fixed cost and variable cost.

#### 3.2.1. Fixed Operator Cost

 Fixed operator cost may not merely depend on the number of running buses but also management and administrative costs. The budget constraint is not considered in this paper, so the fixed costs are not included in the total cost to the operator. We only need to consider the variable cost that is a function of bus travel time and travel distance.

#### 3.2.2. Variable Operator Cost

 The variable operator cost depends on bus travel time and travel distance, including fuel consumption, taxes, maintenance, bus acquisitions, employment cost. *f*_*l*_ is the frequency of line *l* and measured as the number of buses per hour, *CO*_*l*_ is the operation cost per bus online *l*, and *L* is a set of lines on the bus corridor [25]. Then, the variable operator cost *VO* for a set of lines *L* can be expressed as the running cost per hour as follows:

(1) $$\begin{array}{c} VO=\underset{l\in L}{\sum }{CO}_{l}f_{l}\text{.} \end{array}$$

When both running cycle and length of line *l* are considered, the *CO*_*l*_ in (1) is a function depending on the two factors and (1) can be written as (2) and [9]. *R*_*C*_ is the operating cost per bus-hour, *R*_*H*_ is the operating cost per bus-kilometer, *C*_*l*_ is the running cycle of line *l* (hour), and *H*_*l*_ is the total length of line *l* (kilometer) in both directions. The first term in (2) is to calculate the operating cost per bus dependent on bus travel time. It can be expressed as the product of operating cost per bus-hour on a line and the cycle time of the line. The second term in (2) is to calculate the operating cost per bus on a line dependent on bus travel distance of the line. It can be expressed as the product of operating cost per bus-kilometer and the total length of the line in both directions.

(2) $$\begin{array}{c} VO=\underset{l\in L}{\sum }\left(R_{C}C_{l}+R_{H}H_{l}\right)f_{l}\text{.} \end{array}$$

### 3.3. User Cost Considering Fare Payment Modes and Trip Purposes

User cost or passenger cost mainly includes access, waiting, in-vehicle, and transfer time costs. Let us now discuss each of them one by one.

#### 3.3.1. Access Time Cost

 The access time is the time from the start of a trip (say from home) to the moment of arrival at the origin stop plus the time departing from the final bus stop of this trip to the destination (say office or shopping/leisure center). Therefore, the access time depends on the distance between two successive stops. In this paper, the locations of all stops along the corridor of interest are given; hence, the access time is fixed for each trip. It is also assumed that the OD trip matrix is given. Therefore, the access time cost for the limited-stop service problem is constant, which makes it not necessary to include the access time cost in the objective function.

#### 3.3.2. In-Vehicle Travel Time Cost

 The in-vehicle travel time that a user experience in a bus is the running travel time plus all the dwell times at each intermediate stop. The dwell time at a stop includes passenger boarding/alighting times and the door opening/closing time.

The average in-vehicle travel time cost ($) corresponding to a user with trip purpose *j* on route section *s* for an OD pair *w* can be calculated by (3) [26]. *β*_*s*_^*w*^ equals 1 when OD pair *w* travels through section *s,* and 0 otherwise. *P*^*j*^_*TT*_ is the value of in-vehicle travel time savings for a user with trip purpose *j* (*$/h*), *f*_*l*_^*s*^ is the frequency on route section *s* for line *l,* and equal to the frequency of line *l* if line *l* is attractive for route section *s* and 0 otherwise, and *TV*_*l*_^*s*^ is the in-vehicle travel time on route section *s* for line *l* (*$/h*) and composed of the bus running travel time and the time that passengers spend boarding and alighting at intermediate bus stops.

(3) $$\begin{array}{c} TT_{s}^{wj}=\beta_{s}^{w}P_{TT}^{j}\frac{\sum_{l\in L}TV_{l}^{s}f_{l}^{s}}{\sum_{l\in L}f_{l}^{s}}\text{.} \end{array}$$

In this research, we adopt the following form of *TV*_*l*_^*s*^ in (4) given in Larrain and Muñoz [27]. *RT*_*l*_^*s*^ is the travel time on route section *s* for line *l* (hour), and *DT*_*l*_^*s*^ is the sum of average dwell time at all intermediate stops on route section *s* for line *l*. The bus dwell time is a function of the number of passengers boarding and alighting and their average boarding and alighting times which depend on the chosen fare payment mode and the necessary time required to open and close doors.

(4) $$\begin{array}{c} TV_{l}^{s}=RT_{l}^{s}+DT_{l}^{s}\text{.} \end{array}$$

It is also assumed here that the processes of boarding and alighting are simultaneous (different doors to board and alight) and that boarding and alighting flows are independent of each other. Therefore, the dwell time (hour) for a bus at a stop *p* online *l* is the bigger one of passengers boarding and alighting times at the stop (respectively, denoted as *BT*^*m*^_*l,p*_ (hour) and *AT*_*l,p*_ (hour)). The sum of average dwell time *DT*_*l*_^*s*^ is expressed by (5). *P*_*s*_ is the set of the stops on route section *s*, except the stop that the route section *s* arrives at.

(5) $$\begin{array}{c} DT_{l}^{s}=\underset{p\in P_{s}}{\sum }\max\;\left\{BT_{l,p}^{m},AT_{l,p}\right\}\text{.} \end{array}$$

*BT* ^*m*^_*l,p*_ and *AT*_*l,p*_ can, respectively, be given by Eq.(6) and Eq. (7) [24, 27]. *V*_*s*_^*wj*^ is the volume of those passengers (or users) with trip purpose *j* traveling between OD pair *w* on route section *s*, *J* is a set of trip purposes of interest, *t*_*b*_^*m*^ is the average boarding time per passenger (*s*/pax) that depends on the fare payment mode *m*, *t*_*a*_ is the average alighting time per passenger (*s*/pax) that is assumed to a common average value independent of fare payment modes, *W* is a set of OD pairs, and *S*_*p*_^+^ and *S*_*p*_^−^ are two sets of route sections, respectively, departing and arriving at stop *p.*

(6) $$\begin{array}{c} BT_{l,p}^{m}=\frac{1}{3600}\underset{j\in J}{\sum }\underset{k\in S_{p}^{+}}{\sum }\underset{w\in W}{\sum }V_{s}^{wj}\frac{f_{l}^{s}}{\sum_{l\in L}f_{l}^{s}}t_{b}^{m}\text{.} \end{array}$$

(7) $$\begin{array}{c} AT_{l,p}=\frac{1}{3600}\underset{j\in J}{\sum }\underset{s\in S_{p}^{-}}{\sum }\underset{w\in W}{\sum }V_{s}^{wj}\frac{f_{l}^{s}}{\sum_{l\in L}f_{l}^{s}}t_{a}\text{.} \end{array}$$

Integrating Eqs (4)–(7) with (3) gives

(8) $$\begin{array}{c} TT_{s}^{wj}=\beta_{s}^{w}P_{TT}^{j}\frac{\sum_{l\in L}RT_{l}^{s}f_{l}^{s}\sum_{l\in L}\sum_{p\in P_{s}}\max\;\left(\left(\sum_{j\in J}\sum_{s\in S_{p}^{+}}\sum_{w\in W}V_{s}^{wj}f_{l}^{s}/\sum_{l\in L}f_{l}^{s}\right)t_{b}^{m}/3600,\left(\sum_{j\in J}\sum_{s\in S_{p}^{-}}\sum_{w\in W}V_{s}^{wj}f_{l}^{s}/\sum_{l\in L}f_{l}^{s}\right)t_{a}/3600\right)}{\sum_{l\in L}f_{l}^{s}}\text{.} \end{array}$$

#### 3.3.3. Waiting Time Cost

The waiting time is the time having elapsed while passengers are waiting at their origin (and transfer) stop, which is dependent on the bus frequency. A passenger's journey may include multiple stages, referred to as route sections [28], each of which is defined as a fictitious link with a start node, an end node, and a subset of attractive lines serving both nodes. It is assumed that passengers not only do not miss the first arriving bus from the subset of attractive lines serving each route section of their journey but also they take the first arrival bus for their journeys, with an aim to minimize the total travel time incurred.

The average expected waiting time cost for a user with trip purpose *j* bears on route section *s* between an OD pair *w* can be expressed by (9) [26]. *P*^*j*^_*WT*_ is the value of waiting time savings for a user with trip purpose *j*, and *f*_*l*_^*s*^ is a variable taking the value of frequency of line *l* if line *l* is attractive to the route section *s* and 0; otherwise, *k* is a parameter depending on the distribution of bus arrival processes at each stop. When the distribution of bus arrivals is Poisson-distributed, *k* will be equal to 1. However, the bus arrives on schedule, *k* being 0.5. Thus, *k* may be 0.5 to 1 according to the bus arrivals variability [29].

(9) $$\begin{array}{c} WT_{s}^{wj}=\beta_{s}^{w}P_{WT}^{j}\frac{k}{\sum_{l\in L}f_{l}^{s}}\text{.} \end{array}$$

#### 3.3.4. Transfer Cost

The transfer cost should consist of the time passengers spend walking to another stop, the waiting time cost at the stop to take the next bus, and the bus fare for the next bus service. However, in this study, the transfer waiting time cost is not included since it has been considered in the waiting time cost item. For simplicity but without loss of generality, we consider the transfer cost TRANS on the bus along the corridor calculated in (10). *θ*_trans_^*j*^ is the value of the monetary penalty of a user with trip purpose *j* due to transfer, *S* is a set of route sections on the corridor, and *T*_*wj*_ is the volume of those users with trip purpose *j* between OD pair *w*.

(10) $$\begin{array}{c} TRANS=\underset{w\in W}{\sum }\underset{j\in J}{\sum }\theta_{trans}^{j}\left(\underset{s\in S}{\sum }V_{s}^{wj}-T_{wj}\right)\text{.} \end{array}$$

### 3.4. Model with Dwell Times

As passenger flow levels along the corridor rise, the dwell time at a stop may be so large that it can influence passengers' in-vehicle travel times and decisions on passenger route choice. In this case, it is more reasonable to consider the dwell time at a stop directly and, even more precisely, treat it as a function of the number of waiting passengers at the bus stop. Then, the objective function in (11) is to minimize the sum of Eqs (2), (8)–(10), and we have the following line frequency optimization of limited-stop services problem.

In the model, constraint (12) ensures that the frequency of a line on route section is less than the frequency of a line. The constraint set (13) is to ensure passenger flow continuity at each stop. The first term of Eq. (13) makes sure that the sum of passengers of OD pair *w* on all route sections departing from stop *p* is equal to the number of passengers of OD pair *w* if stop *p* is the origin node of OD pair *w*; the second term ensures that the sum of passengers of OD pair *w* on all route sections departing from stop *p* equals the sum of passengers of OD pair *w* on all route sections arrival at stop *p* when the stop is an intermediate stop between the origin node and destination node of OD pair *w;* the third term shows that the sum of passengers of OD pair *w* on all route sections arrival at stop *p* is equal to the number of passengers of OD pair *w* if stop *p* is the destination node of OD pair *w*.

In this case, we may also consider the bus capacity constraints and corridor capacity constraint in Eq. (14) and Eq. (15), respectively. In Eq. (14), the sum of passengers of bus line *l* boarding at and passing through stop *p* is less than the supply of bus line *l* at stop *p.* Eq. (15) makes sure that the number of vehicles on the corridor in an hour is less than the maximum number of buses allowed so as to avoid congestion. The *q*_*l,p*_ is the vehicle capacity of line *l* departing from stop *p*, and *S*_*p*_ is a set of route sections departing from and passing through stop *p*; *P* is a set of stops on the bus corridor. *F*^*p*^_*max*_ is the maximum number of vehicles allowed on the corridor in an hour.

(11) $$\begin{array}{c} \underset{\left\{f_{l},f_{l}^{s}\right\}}{\mathrm{Min}}\underset{l\in L}{\sum }\left(R_{C}C_{l}+R_{H}H_{l}\right)f_{l}+\rho \left[\begin{array}{c} \underset{j\in J}{\sum }\underset{w\in W}{\sum }\underset{s\in S}{\sum }V_{s}^{wj}P_{TT}^{j}\frac{\sum_{l\in L}RT_{l}^{s}f_{l}^{s}\sum_{l\in L}\sum_{p\in P_{s}}\max\left(\left(\sum_{l\in L}\sum_{s\in S_{p}^{+}}\sum_{w\in W}V_{s}^{wj}f_{l}^{s}/\sum_{l\in L}f_{l}^{s}\right)t_{b}^{m}/3600,\left(\sum_{l\in L}\sum_{s\in S_{p}^{-}}\sum_{w\in W}V_{s}^{wj}f_{l}^{s}/\sum_{l\in L}f_{l}^{s}\right)t_{a}/3600\right)}{\sum_{l\in L}f_{l}^{s}}\\ +\underset{j\in J}{\sum }\underset{w\in W}{\sum }\underset{s\in S}{\sum }V_{s}^{wj}P_{TW}^{j}\left(\frac{1}{\sum_{l\in L}f_{l}^{s}}\right)+\underset{j\in J}{\sum }\underset{w\in W}{\sum }\theta_{trans}^{j}\left(\underset{s\in S}{\sum }W_{s}^{wj}-T_{wj}\right)\text{.} \end{array}\right] \end{array}$$

Subject to

(12) $$\begin{array}{c} 0\leq f_{l}^{s}\leq f_{l}\;\forall l\in L,\;\forall s\in S\text{,} \end{array}$$

(13) $$\begin{array}{c} \underset{s\in S_{p}^{+}}{\sum }V_{s}^{wj}-\underset{s\in S_{p}^{-}}{\sum }V_{s}^{wj}=\left\{\begin{array}{cc} T_{wj}\text{,} & ifp=O\text{,}\\ -T_{wj}\text{,} & ifp=D\;\forall p\in P,\forall j\in J,\forall w\in W\text{,}\\ 0\text{,} & otherwise\text{,} \end{array}\right. \end{array}$$

(14) $$\begin{array}{c} q_{l,p}f_{l}\geq \underset{j\in J}{\sum }\underset{s\in S_{p}}{\sum }\underset{w\in W}{\sum }V_{s}^{wj}\frac{f_{l}^{s}}{\sum_{l\in L}f_{l}^{s}},\forall l\in L,\forall p\in P\text{,} \end{array}$$

(15) $$\begin{array}{c} \underset{l\in L}{\sum }f_{l}\leq F_{\max}\text{.} \end{array}$$

The model formulated above is a nonlinear integral model, and we use the branch and bound algorithm coded in LINGO (https://www.lindo.com/index.php/products/lingo-and-optimization-modeling) to solve it.

## 4. Numerical Experiments and Analysis

**Figure 1:**
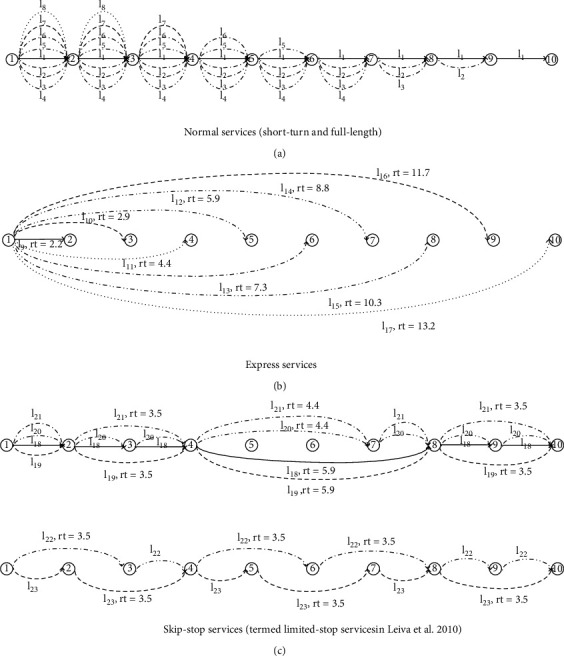
A corridor scenario (Source: Figures 1–3 in (2010)). rt is the travel time on a line section. For instance, *l*_*22*_, rt = 3.5; that is, travel time on a line section connecting stop 1 and stop 3 is 3.5 minutes. (a) Normal services (short-turn and full-length). (b) Express services. (c) Skip-stop services (termed limited-stop services in [9]).

This section applies the models formulated above to the design of limited-stop services on a bus corridor as shown in Figure 1. The corridor settings are the same as in Leiva et al. [9]. It is the segregated lane bus corridor operating along Pajaritos Avenue in Santiago, Chile. Passenger OD demand matrix in Table 1 is taken from Tirachini et al. [30] and covers the one-hour-long demand between each pair of stops on the bus corridor.

As shown in Figure 1, the bus corridor consists of 10 stops which each serves the passenger demand from both directions, and there are 23 alternative lines that serve different stops on the corridor, respectively. It is assumed that the frequency of each alternative line is the same in either direction of the bus corridor, and their services are symmetric in that they visit the same stops in both directions. Leiva et al. [9] divide all 23 lines into three groups, and those serving all stops between the first and the last stops are called normal services.  Figure 1(a) consists of normal short-turn services and normal full-length service (conventional service, line 1), Figure 1(b) shows those express services serving only two stops, and Figure 1(c) illustrates those serving more than two stops and skipping some stops between them and termed skip-stop services in this study (termed limited-stop services in [9]). In fact, short-turn and express services can be regarded as two special cases of limited-stop services if we simply define “limited-stop services” as “services that visit only a subset of the stops on a route.”

It is also assumed that the travel time between two successive stops is 2.2 minutes and that the travel time on a line section that skips at least one stop is listed in Figures 1(b)–1(c). Each of the buses of interest can accommodate up to 80 passengers, but the safety factor often enters into the bus capacity in practice. Here, we assume that the value of the safety margin factor is 0.9; hence, the effective capacity of a bus is 72 passengers, and that passenger average boarding and alighting times are, respectively, 2 s and 1 s with no consideration of fare payment modes [31], and values of travel times are, respectively, 1.5 $/h for in-vehicle time, 3.0 $/h for waiting time, and 0.133$ per person for one transfer that is only as a monetary penalty for taking for an additional bus [9]. The capacity of a corridor is 200 buses per hour without congestion. The operator costs per bus-hour and per bus-kilometer are 6.185$ and 0.375$, respectively [9], and the bus arrivals are Poisson distributed.

## 5. Results

Table 2 shows the empirical advantages of without and with the skip-stop services in the scenario given in Figure 1. It can be seen that the achieved minimum objective function value corresponding to with skip-stop services falls 9.1%, compared to both of normal and express services with no consideration of skip-stop services (see Figures 1(a)-1(b)). Especially, the fleet size saves more about 15.1% considering skip-stop services. It is obvious that passengers' in-vehicle time cost decreases a lot due to fewer stops and higher between-stop speeds with skip-stop services. Moreover, the use of skip-stop services leads to a slight increase in waiting time cost and transfer time cost of passengers. This is because passengers boarding at skipping stops only can be served by regular normal services. Although passengers alighting at skipping stops can be served with all service patterns, partial of those passengers using regular normal services for a direct service need to wait a long time, and others using skip-stop services need to transfer.

**Figure 2:**
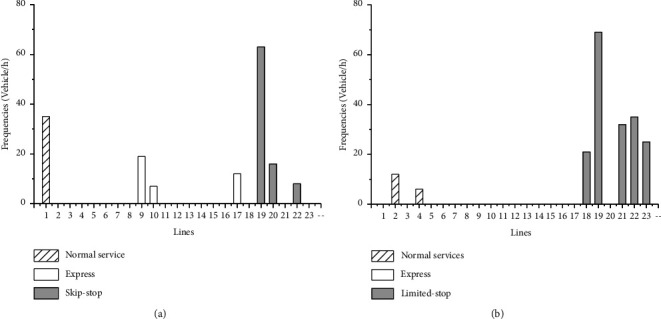
The optimal lines and corresponding frequencies. (a) Service plan without capacity constraints and dwell time. (b) Service plan considering capacity constraints and dwell time.

**Figure 3:**
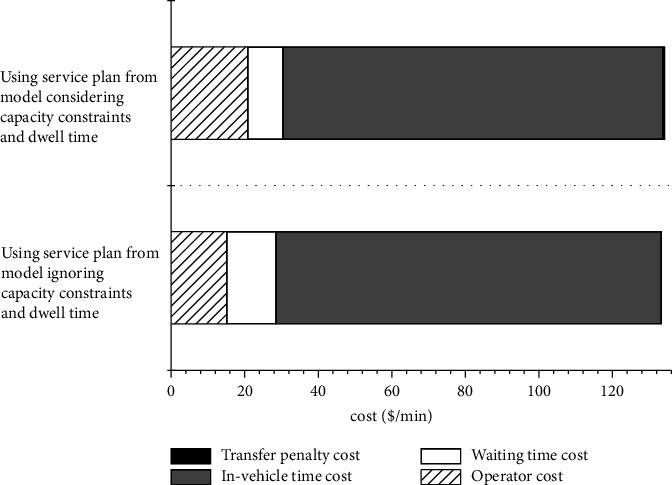
The costs of using two service plans from Figure 2.

The results in Table 3 show that fleet size increases a lot from column 1 to column 4. This illustrates that vehicle capacity and dwell time pay vital roles in design of services. In addition, it is reasonable that the more constraints the larger the OF values are, which is due to the fact that more constraints make the feasible domain of the resulting optimization problem smaller.  Figure 2 shows the optimal service results from two settings corresponding to Table 3 column 1 and column 4. When considering these two aspects, the service plan includes more express and skip-stop services than ignoring them. In addition, it is found that using a service plan from the model ignoring these two aspects (Figure 2(a)) generates more passengers' cost (waiting time, in-vehicle time, transfer penalty) and total cost than a service plan from the model considering them (Figure 2(b)), even though it reduces operator cost due to smaller fleet size (see Figure 3).

### 5.1. Effects of Passenger Demand

**Figure 4:**
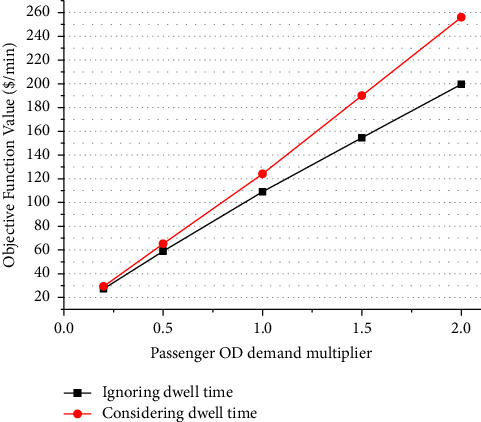
Objective function values under different OD demands.

Figure 4 shows a sensitivity analysis of the objective function value with respect to the passenger OD demand level along the bus corridor. For the convenience of exposition and without loss of much generality, it is assumed that the demand levels of all OD pairs change uniformly; specifically, on the basis of the demand level given in Table 1, they increase or decrease at the same rate that is termed “demand multiplier.” As can be seen in Figure 4, the objective function value rises steadily as the demand level increases, which is true either to the case with or without consideration of dwell times.

The gaps between the two curves in Figure 4 increases as the demand level rises, which implies that the objective function value for the case with consideration of dwell times increases faster than one with no consideration of dwell times. This is not surprising because the latter does not capture the increase in the dwell times at stops and hence increase in the in-vehicle time costs and that in the cycle times. Again, this further illustrates that the degree of underestimation of the total costs to the operator and to the users will increase with the increase of demand if we do not consider the dwell times at stops along the corridor.

### 5.2. Effects of Fare Payment Modes

In the real-life transit system, the fare payment mode choice problem can present a trade-off between the operator's and the passengers' interests. The average estimated alighting times associated with the four different fare payment modes are all 1.42 s/pax (pax short for passenger), and boarding times are 10.02, 4.61, 2.05, and 1.46 s/pax, respectively, for on-board cash payment, magnetic strip, contactless card, and off-board payment [23]. These rapid advances in information and communication technology have made possible using smartphone apps or Internet for payment outside buses, which is a new case of the off-payment mode and has been used in the popular customized bus transit system in China [32].

**Figure 5:**
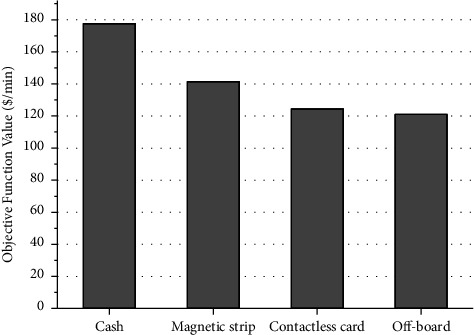
Objective function values of different payment modes.

**Figure 6:**
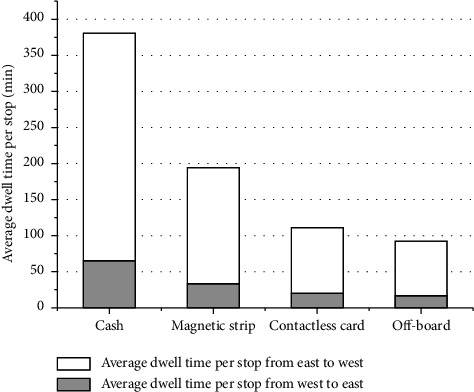
Average dwell time per stop from four payment modes.

Figure 5 displays the values of the objective function (i.e., the total costs to the bus service provider and to the passengers) when each of the four fare payment modes is implemented. It shows that, among all four payment modes, the cost incurred due to the use of the on-board cash payment mode is the highest, and the cost arising from off-board collection is the lowest. This is because the on-board cash payment mode requires passengers get their money or get their big notes changed to coins first, which can largely lengthen the bus dwell times at stops. As shown in Figure 6, the average dwell time per stop to cash payment mode is the highest one in investigated period. It is obvious that the off-board payment mode is the most efficient, saving around 33% cost compared to the on-board cash payment mode.

**Figure 7:**
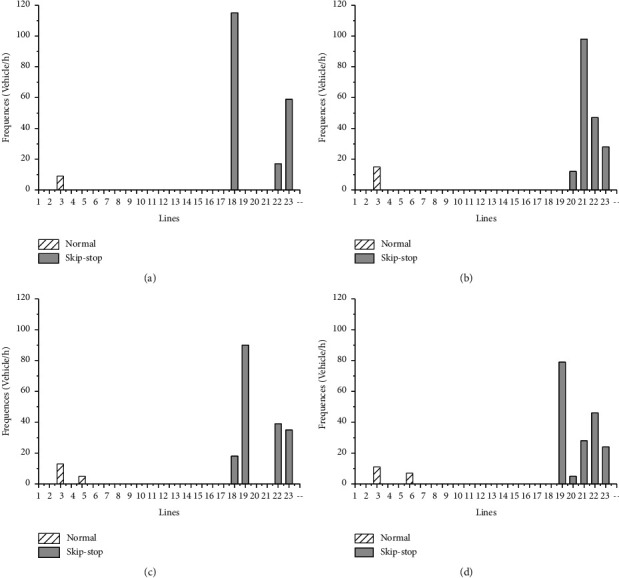
Frequencies of optimal lines given by four payment modes. (a) Cash. (b) Magnetic strip. (c) Contactless card. (d) Off-board.

**Figure 8:**
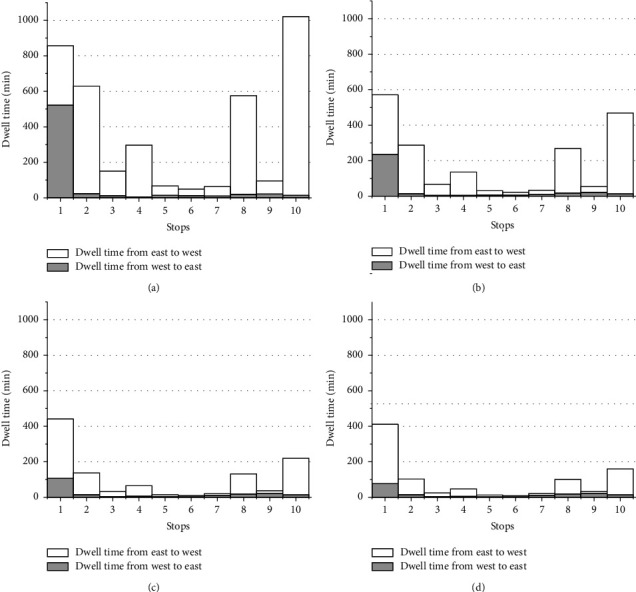
Dwell time at each stop from four payment modes. (a) Cash. (b) Magnetic strip. (c) Contactless card. (d) Off-board.

Figure 7 lists and compares the frequencies of the resulting bus lines corresponding to the four fare payment modes. It indicates desirability of skip-stop service and relatively low the number of optimal lines when cash payment mode is provided, since skip-stop service saves more time when payment system is slower. Different combinations of optimal lines should be offered for transit system in terms of type of payment mode. The dwell time per stop generated by four modes in Figure 8, off-board, and contactless card relatively save more dwell time at stop, especially at stops with high passengers boarding or alighting.

### 5.3. Effects of Trip Purposes

The values of passenger travel time can vary quite a lot as passengers' trip purposes differ, such as leisure, shopping, commuting, and business. In general, business and commuting trips have higher values of travel time than those trips with other purposes.  Table 4 shows a group of estimated values of travel time with different purposes [33]. In addition, a common rule is that the value of waiting time is considered twice as the value of in-vehicle time, which has been supported by several studies reviewed by Wardman [33].

In addition, the proportion of users with a given trip purpose in passenger flows varies in different periods of a day. Most of passengers travel for commuting in the morning or evening rush period while most of passengers travel for other purposes such as leisure or shopping that happen in the off-peak period.  Table 5 shows a set of proportions (weights) associated with different trip purposes in different time periods of a day.

**Figure 9:**
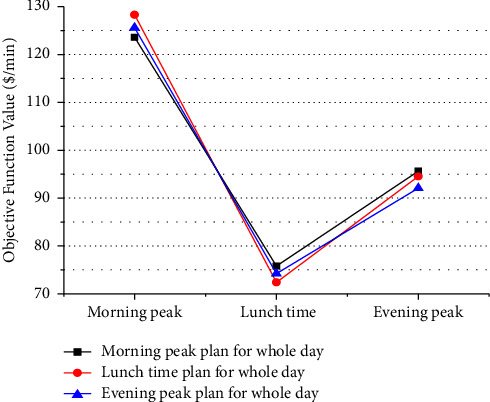
Objective function under different limited-stop service plans.

Figure 9 shows an empirical analysis of influence of different users with different values of time. The horizontal axis shows three important time periods of a day for bus services. Passengers are composed of multiclass passengers with different trip purposes at different periods. As can be seen in Figure 9, on assumption of fixed OD demand matrix in different periods, the social total cost is not always least if we took the same stop service plan for whole day. For example, in the morning peak period, if we took the stop service plan for lunch time or evening stop services, the total social cost will be higher than the optimal stop service plan for the morning peak period. Although the stop service plan for lunch time or evening peak uses fewer vehicles to decrease the bus operator cost, it would, however, increase the user costs due to longer travel times. In practice, the difference will be more apparent because generally, passenger flows in off-peak period are lower than that in peak periods, and the total frequencies of bus lines given for off-peak period will be less than that for peak period. Therefore, passengers would spend more time for their travel if the stop service plan for off-peak period is used for peak period. Hence, it is very important to consider multiclass passengers in the proposed model for optimizing the limited-stop bus services in the corridor by time of day.

### 5.4. Sensitivity Analysis of Balance Parameter *ρ*

**Figure 10:**
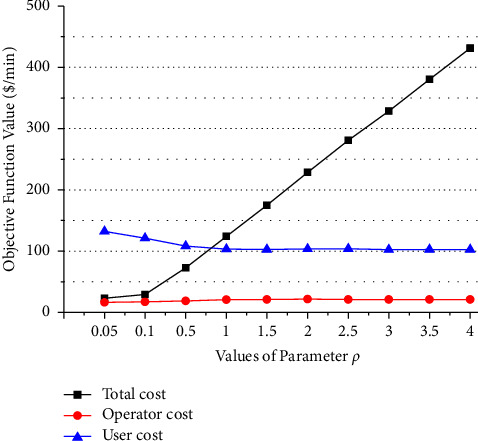
Sensitivity analysis of parameter.

Figure 10 presents a sensitivity analysis for assessing the impacts on operator cost and user cost of the newly proposed balance (or weighting) parameter *ρ* in the objective function (operator cost + *ρ* user cost). It shows that, as the value of the parameter increases, the user cost declines, and the operator cost rises till to be constant. The model is linear with relation to parameter *ρ*. So when the total frequency of lines is up to the maximum value, there is no change in user cost and operator cost with increase of *ρ*. The planner should adjust carefully the parameter value to balance the interests of the bus operator and users. The larger value of the balance parameter and no more than a threshold may be used in the optimization model if the planner wants to provide subsidy for passengers to travel using buses. On the contrary, the smaller value of the weighting parameter could be used if the planner wants to encourage the bus service operator to provide better services.

## 6. Concluding Remarks

This paper proposes an optimization model for the design of urban limited-stop bus corridor services with consideration of fare payment mode choice and passenger trip purposes. In the proposed model, the cases with or without dwell time at a stop are also considered with bus capacity constraints. The objective function of the proposed model is a weighted sum of the costs to the bus service provider and to the passengers, and the weight is a parameter for the planner to balance the interests of the operator and the passengers. The effects of fare payment mode choice are captured by the dwell time at a stop, and the parameters for the values of travel time savings are used to reflect passenger trip purposes. This largely theoretical model for optimization of bus routes within a corridor is the key contribution of this paper to the design of limited-stop bus corridor services.

The numerical analysis presented provides a comparison of the effects of differing choices on service patterns and frequencies, rider payment methods, etc. In the numerical experiments, the sensitivity of the total cost to the passenger demand level is investigated, which shows that the necessity of consideration of bus capacity constraints as well as dwell times when the passenger demand level is high. It was found that ignorance of dwell times and bus capacity constraints at stops would underestimate the cost to the service operator and the cost to the passengers. When the effects of fare payment mode choice are analyzed with the use of the proposed model, it was suggested that the bus operator should be encouraged to choose the off-board fare payment mode (e.g., smartphone apps/Internet used in customized bus transit system) and contactless cards.

The adoption of a fare payment mode is subject to how much time that might be saved and how much investment is required to introduce the fare payment system. This may be a straightforward cost-benefit analysis. As discussed previously, we can readily work out how much time a new fare payment system may help to save. Then, we can work out the savings of passengers' travel time costs. If these savings are higher than the cost incurred due to the adoption of the new system, then it is acceptable to introduce the new payment system.

A number of insightful findings derived from the application of the proposed model have been drawn from the numerical results:

- The total operator and user cost obtained by skip-stop services saves 9.1% over the normal and express services in the corridor.
- There is a noticeable difference in the social cost between the case with capacity constraints plus bus dwell time at intermediate stops and the case without them. It was found that the bus dwell times have a greater influence on the total cost than the bus capacity constraint.
- Compared to on-board fare payment modes, off-board payment modes are more efficient. The adoption of off-board payment modes can reduce the total social cost since the off-board payment mode collects the fare out of vehicle and reduces passengers' boarding times at stops. In other words, the off-board payment mode reduces the bus dwell time at a stop. Cash payment system makes skip-stop and expresses services more preferable because skip-stop activities save more time when payment system is slower.
- It is not desirable to apply the same limited-stop service plan to any period of a day because an optimal plan for the morning period is highly likely not to be optimal any more for the evening period.
- The planner can adjust the value of the balance parameter to encourage the operator to provide better bus service, or attract more passengers to travel using buses due to lower travel cost, or find a balance point that would satisfy both the operator's and users' interests.

One question that may occur is the feasibility of using the proposed approach in real life and how useful it could be for transit agencies. We answer this question from five aspects: (i) Are the proposed solutions for the problem of interest too complicated for users/passengers to understand? It may be a bit too optimistic to believe that passengers can understand a set of 4 to7 different lines for traveling along a corridor. Moreover, in practice, we may paint the buses along the corridor in different colors so that passengers can find their buses conveniently. For instance, a blue color means the use of a short-turn service. Nowadays, we may use the smartphone, Internet, and variable message signs on board to display the arrival times and service patterns of buses in real time. In addition, once passengers get used to such schemes of multilines/routes sharing one corridor, their benefits due to less journey times will outperform the confusion some may feel while traveling along this corridor. Certainly, the number of lines/routes along a corridor should not be too many just in case passengers get confused in choosing which line/route to take. It is believed that there should be a critical value for this number that may be dependent on the distance between the first and last stops and identified by stated or revealed preference surveys or other experiments. (ii) Do passengers always take the first bus that arrives, in particular when certain express buses are available? It may be widely acknowledged that it is a very strong assumption believing that passengers will take the first bus to arrive at stops but it may be true in the average sense. In addition, this assumption gives us a tractable model for the problem of interest. (iii) When is fare payment a relevant or a critical design variable? For the corridor of interest in this paper, having a segregated lane for buses may be more important than having off-board payment. It shall be safe to say that the adoption of off-board payment facilities is cheaper than the implementation of exclusive segregated lanes. Moreover, it has made possible using smartphone apps or Internet for payment outside buses, which has been used in the popular customized bus transit system in China. Therefore, maybe fare payment can be an alternative to the special lanes at certain demand levels. (vi) When shall we start thinking about changing fare payment methods on a corridor? This is out of the scope of this paper but the answer for this can be as simple as the outperformance of the alternative fare payment mode over the current one due to the potential value of time savings for all passengers, which is higher than the cost incurred. (v) How far may a passenger be willing to walk for a transfer to another line/routing time (access time). Passengers can decide walking to further stop in order to take a more convenient route (this is very common in real life). The distance a passenger walks for transfer may be longer or shorter than the gap between two stops. The specific value of the critical distance may vary in different areas of a city or in different cities (and countries).

While sensitive to many relevant factors such as time, payment methods, type of service pattern, the proposed model in this paper requires a specific origin–destination matrix for optimization. Additionally, the matrix must be stratified by trip purpose for further refinement of optimal service patterns. A matrix stratified by time of day and trip purpose is a next forwarding move of research on this topic.

It is noteworthy that the key intellectual merit of this paper is not these new findings but the proposed model that can handle the problem of limited-stop bus corridor service design with the consideration of fare payment mode choice and trip purposes. An ongoing piece of our research on this topic is that demand is elastic plus the effects of stochasticity of travel times between two stops on optimal service schemes. Moreover, in future studies, our methodology has the potential to examine transit networks using limited-stop services and to analyze the real-life transit systems around the world considering built environment characteristics such as bus stop accessibility. Personalized recommendations and guidance could be provided to passengers to use the limited-stop services [34]. Last but not least, the limited-stop services can be integrally optimized with vehicle scheduling.

## Figures and Tables

**Table 1:** OD matrix (hourly passengers) on the bus corridor (Source: Tirachini et al. [30]).

D	1	2	3	4	5	6	7	8	9	10
O										
**1**	0	600	189	165	64	44	342	605	726	395
**2**	3620	0	11	10	4	3	20	35	42	23
**3**	790	38	0	5	2	1	10	18	22	12
**4**	1585	75	82	0	0	0	2	4	5	3
**5**	281	13	14	14	0	2	13	24	29	16
**6**	186	9	10	9	8	0	13	22	27	15
**7**	264	13	14	13	12	9	0	12	14	8
**8**	2631	125	135	130	117	86	107	0	36	19
**9**	337	16	17	17	15	11	14	18	0	67
**10**	4425	211	228	218	197	144	180	232	200	0

**Table 2:** A comparison of bus stop services without and with skip-stop services.

Items	Without skip-stop services (Figures 1(a)–1(b))	With skip-stop services (Figures 1(a)–1(c))	Change (%)
Obj. Fun. Value ($/min)	136.5	124.1	**−9.1%**
Total operator cost	23.2	20.8	**−10.3%**
Fleet size (no. of buses)	146	124	**−15.1%**
Frequencies (buses/h)	200	200	**—**
Total cost to the passengers	113.3	103.3	**−8.8%**
Waiting time cost	5.2	9.7	**—**
In-vehicle time cost	108.1	93.2	**−**13.8%
Transfer cost	0.00	0.4	**—**

**Table 3:** Values of objective function (OF) with different settings.

Items	Neither capacity constraints nor dwell time	With capacity constraints but without dwell time	Without capacity constraints but with dwell time	With capacity constraints and dwell time
OF value ($/min)	106.0	109.1	122.9	124.1
% Rise in OF	—	3%	16%	17%
Total operator cost	13.4	18.7	20.4	20.8
Fleet size (no. of buses)	76	106	121	124
Frequencies (buses/h)	159	200	200	200
Total cost to the passengers	92.6	90.4	102.5	103.3
Waiting time cost	13.5	10.3	9.4	9.7
In-vehicle time cost	79.1	80.0	92.9	93.2
Transfer penalty cost	0.0	0.1	0.2	0.4

**Table 4:** Values of travel time of multiclass users with different trip purposes.

Trip purpose	Value of in-vehicle time ($/h)	Value of wait time ($/h)	Value of transfer ($/h)
Leisure	0.833	1.667	0.067
Shopping	1.000	2.000	0.100
Commuting	1.500	3.000	0.133
Business	1.833	3.667	0.167

**Table 5:** Weights for different trip purposes in different periods of a day.

Period	Leisure	Shopping	Commuting	Business
	Weight of users	Weight of users	Weight of users	Weight of users
Morning peak	0.1	0.1	0.7	0.1
Lunch time	0.43	0.42	0	0.15
Evening peak	0.2	0.2	0.5	0.1

## Data Availability

All data used to support the findings of this study are included within the article.

## APPENDIX

Notations include sets, parameters, state, and decision variables.

### A. Sets

*L*: set of bus lines
*W*: set of OD pairs
*S*_*p*_^+^: set of route sections departing at stop *p*
*S*_*p*_^−^: set of route sections arriving at stop *p*
*S*: set of route sections on the corridor
*J*: set of trip purposes of interest
*S*_*p*_: set of route sections related to stop *p*
*P*: set of stops on the bus corridor
  Ps: set of the stops on route section *s*, except the stop that the route section *s* arrives at

### B. Parameters

*R*_*C*_: vehicle operating cost per hour ($/vehicle.h)
*R*_*H*_: vehicle operating cost per distance ($/vehicle.km)
*C*_*l*_: running cycle of line *l* (h)
*H*_*l*_: total length of line *l* (km)
*β*_*s*_^*w*^: binary parameter, equals 1 when OD pair *w* travel through section *s* and 0 otherwise
*P*^*j*^_*TT*_: value of in-vehicle travel time savings for a user with trip purpose *j* ($/h)
*P*^*j*^_*WT*_: value of waiting time savings for a user with trip purpose *j* ($)
*t*_*b*_^*m*^: the average boarding time per passenger that depends on the fare payment mode *m* (*s*/pax)
*t*_*a*_: the average alighting time per passenger that is assumed to a common average value independent of fare payment modes (*s*/pax)
*q*_*l,p*_: the vehicle capacity of line *l* departing from stop *p*
*π*_l_^p^: binary parameter that equals *1* if the line visit stop *p* and 0 if it does not
*F*^*p*^_*max*_: the maximum number of vehicles to stop *p* in an hour
*θ*^*j*^_*trans*_: the value of the monetary penalty of a user with trip purpose *j* due to transfer
*k*: a parameter depending on the distribution of bus arrival processes at each stop
*ρ*: a weighting parameter adopted to balance the operator cost and the passenger cost

### C. State variables

*VO*: variable operator cost
*CO*_*l*_: operation cost per bus online l
*TT*_*s*_^*wj*^: the average in-vehicle travel time cost corresponding to a user with trip purpose *j* on route section *s* for an OD pair *w* ($)
*TV*_*l*_^*s*^: in-vehicle travel time on route section *s* for line l
*RT*_*l*_^*s*^: travel time on route section *s* for line *l* (h)
*DT*_*l*_^*s*^: the sum of average dwell time at all intermediate stops on route section *s* for line *l* (h)
*BT*^*m*^_*l,p*_: total passenger boarding times at a stop *p*online l
*AT*_*l,p*_: total passenger alighting times at a stop *p* online l
*V*_*s*_^*wj*^: the volume of those passengers (or users) with trip purpose *j* traveling between OD pair *w* on route section s
*WT*_*s*_^*wj*^: the average expected waiting time cost for a user with trip purpose *j* bears on route section *s* between an OD pair *w* ($)
*TRANS*: the transfer cost on the bus along the corridor ($)
*T*_*wj*_: the volume of those users with trip purpose *j* between OD pair *w*

### D. Decision variables

*f*_*l*_: frequency of line *l* (vehicle/h)
*f*_*l*_^*s*^: frequency on route section *s* for line *l*, and equal to the frequency of line *l* if line *l* is attractive for route section *s* and 0 otherwise
